# The Requisites of Food

**Published:** 1890-11

**Authors:** 


### THE REQUISITES OF FOOD.

In general terms it may be said that the presence of life is conditioned upon the exercise of the functions of absorption and assimilation of various food materials. When a living organism is under normal conditions, these functions maintain its physical integrity, and enable it to go through its cycle of development in accordance with its physiological law ; when they have finally discontinued their operations the organism perishes. By the presence or absence of these functions, therefore, we can easily distinguish between organic and inorganic substances, a distinction by the way which has been made since the earliest times. All living substances, during their period of existence, therefore, demand regular supplies of food material in form and character suitable for absorption and assimilation.

Among the lowest forms of life, we observe the greatest simplicity in this respect ; indeed the rule is that only one kind of food or pabulum will support the organism and permit its growth. Thus we have well known forms of micro-organisms that develop only in certain chemical solutions, such as the *micrococcus urœ*, causing the ammoniacal fermentation of urine, the micrococcus of mucoid wine fermentation, the micrococcus causing phosphorescence in putrid meat and fish ; the bacterium lactis causing lactic acid fermentation in milk, and the bacterium aceti associated with acetic acid fermentation. Advancing in the scale we find nearly every variety of vegetable product accompanied by a parasite to which it affords the appropriate food. Even the higher animals in their natural state have a very limited variety of articles from which to select, and which affords them all the nourishment needed for their growth. In a state of domestication, the effects of different kinds and of greater variety of foods have been carefully studied, both physiologically and economically, and biological science has been enriched by the results of innumerable investigations, since Boussinggault first opened this important field of investigation. At the present time, stock raisers know how to feed so as to produce fat, or on the other hand so as to increase muscle and bone. Special traits of form or color may thus be cultivated, and in fact, according to Darwin, all the existing varieties of domestic animals are the result of man's controlling agency, especially in the direction of methodical selection and feeding. These facts are common knowledge, but they demonstrate the far reaching and important relations of food, and are suggestive in regard to the diet of man.

One distinguishing feature by which the dietetic problem in man differs widely from that of the lower animals is the absence in the latter of the gastronomical element. The animal eats its appropriate food with relish, and as long as it thrives seeks no change from year to year and from generation to generation ; man, on the contrary, in his civilized state, demands an extended dietary, and tires of even the most wholesome and appetizing articles, even acquiring disgust for them if too long continued. The anecdote of "*toujours perdrix*" (partridge every day), is here very much to the point. We observe, however that the animals nearest to man, living upon comparatively very few articles of food, are very rarely troubled by any thing like indigestion.—*Ex. from The Dietetic Gazette.*

